# Enhancing Youth Participation Using the PREP Intervention: Parents’ Perspectives

**DOI:** 10.3390/ijerph14091005

**Published:** 2017-09-02

**Authors:** Dana Anaby, Coralie Mercerat, Stephanie Tremblay

**Affiliations:** 1 School of Physical and Occupational Therapy, McGill University, Montreal, QC H3G 1Y5, Canada; stephanie.tremblay2@mail.mcgill.ca; 2 Department of Psychology, Université du Québec à Montréal, Montréal, QC H2X 3P2, Canada; coralie.mercerat@gmail.com

**Keywords:** participation, youth, childhood disabilities, community, leisure

## Abstract

Pathways and Resources for Engagement and Participation (PREP), an innovative intervention aimed at modifying the environment and coaching youth/parents, was found to be effective in improving youth participation in chosen community activities. In order to complement existing quantitative evidence, this study examined parents’ perspectives on the PREP approach. Twelve parents of youth with physical disabilities (12 to 18 years old) who received the PREP approach participated in individual semi-structured interviews following the 12-week intervention delivered by an occupational therapist. Thematic analysis revealed three inter-linked themes, the first of which was informative, describing the “nature of intervention”, and led to two reflective themes: “multi-faceted effects of care” and “process of care”. Parents highlighted the effect of the PREP intervention in a broad sense, extending beyond the accomplishment of the selected activities. This involved improvements on the physical, emotional, and social levels as well as in autonomy. Parents also discussed how their own needs were acknowledged through the intervention and recognized the unique role of the occupational therapist in supporting this process. The findings provide additional information about the usefulness of the PREP approach and describe the various benefits generated by a single intervention. Such knowledge can expand the therapeutic options for positive, health-promoting participation.

## 1. Introduction

Participation of children and youth in leisure community-based activities is key to their health and well-being [1,2] and is considered one of the most important outcomes in pediatric rehabilitation [3], acknowledged by both clinicians and parents [4,5]. Through participation, youth can improve physical health and fitness, psychosocial well-being, academic achievement and reduce risk-taking and problem behaviors [6,7]. The participation of youth with disabilities, however, is restricted in comparison to their typically developing peers [8,9] which can lead to poor health outcomes, making the challenging transition to adulthood even more complex [10,11]. To date, little is known about effective therapy interventions for enhancing the outcome of participation [12]. It is therefore important to develop new intervention strategies for improving youth participation as well as test their effectiveness from various perspectives. 

The PREP (Pathways and Resources for Engagement and Participation) [13] approach is an innovative strengths-based intervention aimed at enhancing participation in chosen community-based leisure activities by removing environmental barriers and coaching youths and their parents. This 12-week client/family-centred, customized intervention involves five steps: (1) Make goals; (2) Map out a plan; (3) Make it happen; (4) Measure process and outcomes; (5) Move forward. It is delivered by an occupational therapist (OT). Specifically, an OT met with each youth and their family in their home. Together, they identified three participation goals or community-based activities in which the youth wanted to participate, yet found difficult to engage in. Examples of activities included playing boccia, riding a bike, joining a youth club or a sledge hockey team. They then devised a plan to minimize barriers within the environment and build on supports so that the youth could participate in three activities of their choice. Examples of environmental barriers included physical inaccessibility, unsuitable activity equipment, unavailability of programs, poor access to information and transportation, lack of knowledge within community agencies about ways to adapt their programs/provide accessible services, unsupportive attitudes of others, and limited social support. The PREP approach has demonstrated evidence of effectiveness in improving levels of performance in meaningful community-based activities [14,15] and was positively perceived by OTs who had implemented it [16]. Findings from a recent intervention study examining the PREP approach among 28 youth with physical disabilities revealed a clinically and statistically significant improvement (*p* < 0.0001) of more than 2 points on the Canadian Occupational Performance Measure scale across 79 activities/participation goals set by the youth [17]. While considerable quantitative evidence of the effectiveness of the PREP approach based on youth self-perceived performance exists, it is important to complement these findings with the parent’s perspective on the impact of the intervention. Such qualitative inquiry will result in a comprehensive understanding of the nature, process and outcomes of intervention, and can further inform the implementation of the PREP approach in practice while taking into account parents’ views and expectations. This is of particular importance because parents’ beliefs regarding the credibility and effectiveness of therapy interventions are linked to treatment adherence and, consequently, the child’s outcomes [18]. The purpose of this post-intervention qualitative study was therefore to elicit and explore parents’ perceptions and experiences of the PREP approach. 

## 2. Materials and Methods

Twenty-two youth aged 12 to 18 years who have mobility restrictions (e.g., due to cerebral palsy, spina bifida, musculoskeletal disorders) were recruited from five major rehabilitation centers and two high schools in Greater Montreal, from both the Anglophone and Francophone communities. Adolescents who were recovering within the first year following a severe brain injury were excluded, as their functional capacities are less likely to be stable. A convenience subsample regrouping twelve parents of twelve (out of 22 eligible) youth with physical disabilities who received the PREP intervention were asked to participate in an interview and provided informed consent. Sampling continued until data saturation was reached (no new codes came up during the analysis). Two to four weeks following the intervention, individual semi-structured interviews lasting up to one hour were conducted with each parent at their home by one of 3 interviewers, two of which were not involved in the intervention portion, and the third one having had no interaction with their interviewees during intervention (i.e., worked with other families). The interview included 5 open-ended questions in addition to follow-up prompts and was validated by two experts to reduce ambiguity and leading questions. The interviewers began by asking the parents to describe their general experience of the intervention. Information was also elicited about specific aspects such as barriers and strategies related to their child’s participation in their chosen activities, as well as effective and ineffective elements of the intervention. Interviews were audio-recorded and transcribed verbatim. All information that might identify a participant was encrypted and aliases were used to ensure confidentiality. Ethics approval was obtained by the Centre for Interdisciplinary Research in Rehabilitation of Greater Montreal (CRIR-865-0713).

Data analysis: The content of each interview was coded using the QSR-International NVivo11 software (QSR International Pty Ltd., Victoria, Australia) and analyzed following the six-step thematic analysis outlined by Braun and Clarke [19]. In order to gain a deeper understanding of the findings, the principal researcher and research assistants took part in the collection, transcription and reading of the data. Then, two research assistants, including one independent of the intervention, autonomously coded the first four interviews and generated an initial coding scheme (by bringing different codes together, in order to create themes). Following this step, the research assistant who was independent of the intervention continued to code the remaining 8 interviews and refined the themes and subthemes in collaboration with the principal researcher. Hierarchy across themes and sub-themes was discussed to create a thematic map through on-going meetings, and themes were identified and named in order to further support the reliability of the findings [19]. The principal researcher and the independent research assistant met frequently to build the narrative frame of the findings. Throughout the six stages, themes were checked back with the original data and vice versa by reading and re-reading the collated extracts for each theme as well as the entire data set to ensure that the thematic map reflected the meaning evident in the data set as a whole. This process was supported by two team members, both clinical researchers, who provided input about the description and the interpretation of the data [20]. 

## 3. Results

### 3.1. Sample Characteristics

12 parents of youth with physical disabilities participated in the study. This subsample was not significantly different from the rest of the group (*n* = 22) in terms of age (t = −0.66, *p* = 0.517) and gender (χ^2^ = 0.733, *p* = 0.392), alleviating potential selection bias. Table 1 describes the parents’ demographic characteristics. The youth, who ranged from 12 to 18 years old (mean = 14.3, SD = 2.1), presented with a variety of health conditions (ranging from 1 to 5, mean = 2.6) including speech/language impairments (58%), orthopedic/movement difficulties (58%), developmental/intellectual delay (33%) and visual impairments (25%). They also had a number of functional issues (1 to 11, mean = 5.1) such as difficulty using their hands to do activities (92%), communicating with others (58%) and moving around (50%). The majority of the youth (*n* = 10) attended special classes/schools.

### 3.2. Emerging Themes

**Figure 1:**
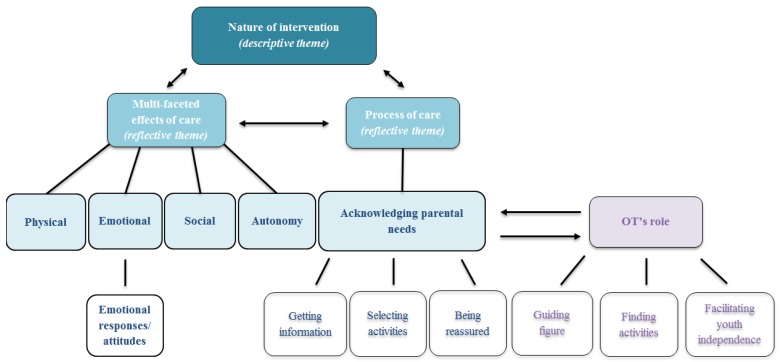
Structure of themes generated by the data.

Three main inter-linked themes emerged from the data: nature of intervention, multi-faceted effects of care and process of care (see Figure 1). The first (nature of intervention) was essentially descriptive since parents were asked to provide information about specific elements or steps of the intervention and their responses extended beyond the explicit questions. This descriptive theme, illustrating the focus and modality of the intervention and its distinct characteristics meaningful to parents, generated two additional reflective themes. One was the multi-faceted effects of care, which refers to parents’ perceptions of the intervention’s impact on their child on different levels (e.g., physical, emotional, social) and the other was process of care, indicating the parents’ specific needs (e.g., getting information, selecting activities, being reassured) and the way they were (or were not) addressed within the intervention. Addressing parental needs was intrinsically related to the roles of the OT. 

### 3.3. Nature of Intervention

Parents described a range of individualized barriers that were addressed in the intervention with a special focus on the unique way that the intervention was delivered. The intervention was customized to the youth, parents and family characteristics as well as to their logistical needs in terms of the planning and management of daily schedules. Time management was frequently recognized as a barrier to participation in various activities. For example, parents reported how busy and “unpredictable” their daily routine was, especially when there were other children—and therefore different schedules—within the family. They acknowledged two elements or characteristics of the intervention which alleviated time management issues. These involved the OT meeting the youth, usually every week, at the family’s home, minimizing parents’ commute time. Facilitating youth participation in activities within their natural and immediate environment was another supportive element of the intervention, as illustrated by Laura’s mother when describing the scrapbooking class she started attending: “For us, it was important that we stayed around. I didn’t want to have to drag Laura into traffic for the classes. Yes, so (the OT) found the perfect place for us.”.

Financial constraints were likewise frequently identified as a barrier to participation. Dedication of the therapist to find affordable activities and the funding support provided by the study were recognized by parents as a way to overcome this obstacle. In addition, barriers within the environment (e.g., physical, social and attitudinal) were highlighted. Re-structuring the physical space in which the activity occurred, for instance by changing the layout of the room and adjusting the sitting position to enable participation in a drawing class, and educating the youth and the on-site instructor about modifying the environment to make it suitable were described by parents as one of the main helpful strategies used by the OTs. 

Parents also discussed how the intervention was customized to the youth’s characteristics (e.g., physical abilities/health related conditions, motivation or personality traits) and was focused on their chosen activities. In fact, more than half of the parents (*n* = 8) identified that the goals were tailored to the child’s interests and abilities, as underlined by Patrick’s mother: “Ah, that was excellent because the OT, she discussed with Patrick to know what he wanted. […] She helped a little to start thinking, but they were really his ideas” (for example to participate in power wheelchair basketball). The therapist evaluated the youths’ strengths and weaknesses before trying an activity and re-adjusted the goals based on their abilities. OTs were particularly supportive in giving youth the opportunity to overcome barriers on their own. Jessica’s mother described the way the OT showed her daughter different ways to accomplish the activity she wanted to do (walking two dogs simultaneously while holding the leashes in a particular way to make it easier). Similarly, the OT suggested to Eric and the instructor of his church club a new way to re-arrange the room’s layout in order to allow Eric to navigate the space using his wheelchair: “He is starting to feel that he has his place there, that he can participate as much as anyone else, you know. So, I think that… I think [the OT] was able to transmit the strategies to Eric effectively [and now] he does it almost every week”. These strategies led to success in achieving goals and had a globally positive effect on the youth, as described in the following theme. 

### 3.4. Multi-Faceted Effects of Care

Overall, the intervention resulted in generally positive experiences which led to a wide array of favorable outcomes. The majority of parents expressed feelings of success related to the intervention as the youth were able to accomplish their chosen leisure and participation goals. Furthermore, success was expressed across four different levels, namely physical improvement, emotional growth, social expansion and greater autonomy. For example, David’s mother observed progress at both the autonomy and social levels: “Yes, he took the bus alone with his friends to come home. He had an exam, he finished early, and he took the city bus back home with his friends, so that’s a barrier that we’re overcoming, you know? […] So it’s happening, things are slowly coming along.”. 

Parents described how the intervention led to improvement at the physical level, although this was not specifically targeted by the OT. Overall, it appeared that the youth were more capable of accomplishing tasks and participating in activities in which they had difficulty with prior to the intervention (e.g., dancing, practicing fine motor skills, using the left hand more). Lisa’s father commented on his daughter’s goal of participating in dance classes: “so on the physical level […] that is only beneficial, it was nothing but benefits for her […] she won’t be at the same rhythm as the others, but at least she can go. To be able to express herself in that domain.”.

Participants’ progress was also observed in terms of emotional growth; this involved the notions of pleasure, enthusiasm towards activities and a change in attitudes/emotional responses exhibited by greater self-esteem and confidence. Patrick’s mother observed an overall improvement in her son’s mood, particularly related to his motivation. This change had a significant impact on his mother, as she “felt better” seeing her son participating in activities despite the winter season. This was a rewarding experience for both the parents and the participant. The notion of pleasure was expressed by several parents, as the participants displayed enthusiasm towards the activities and their ability to accomplish them. To illustrate, David’s mother described her own feeling of happiness, seeing her son enjoying the activities (especially luge hockey): “I’m happy. He enjoys it. He loves going, and I see he enjoys doing stuff, and it’s just, as a parent, you see your kid enjoying an activity, it’s something, you know.”. Jessica’s mother observed a difference in her daughter’s attitudes and emotional responses. She wanted her daughter to “get up and do something” which previously led to some conflicts between them. Following the intervention, her daughter accomplished her goals of walking dogs, shopping, and skiing. This resulted in a change in attitude of the young girl. The mother felt that her daughter gained greater awareness, motivation, and determination, declaring her daughter reframed herself as a person who doesn’t “want arthritis to prevent [her] from living or doing things”. Moreover, this experience was reported to have a positive impact on the mother-daughter dynamic as the mother asserted: “I would say [that it has had] a rather positive impact, in terms of the level of conflicts and the perspective we parents now have [toward her]”. 

The effect of the intervention also involved a process of a social expansion, as the youth initiated and participated in new, additional activities in the community. These activities, not necessarily targeted by the intervention, involved making new friends (thus being more accepted by others) and taking on new roles (e.g., becoming a member of a social committee, participating in a play at the school theatre). Finally, the notion of autonomy was also described as a success for parents, the youth and the therapist. Laura’s mother perceived her daughter as less dependent on her, meaning she could engage in the community without her mother. For Patrick’s mother, autonomy was achieved when her son decided to schedule his transportation by himself to go to a certain activity. These skills extended beyond the specific participation goal or activity, as Patrick can now organize most of the adaptive transportation services on his own, for example to go to the mall. 

### 3.5. Process of Care

Along with the effects on the youth, the process of the intervention was seen by the parents as a facilitator in fulfilling their own needs. Three main parental needs emerged relating to their child’s participation in activities: getting information [about resources and available options], selecting activities, and being reassured. The OT role emerged as a supportive and guiding figure, interwoven in parents’ discourse about acknowledging their expressed needs. 

Getting information related to the possible activities and resources available in their community was a parental need often addressed in the intervention. Lisa’s father underlined the lack of information, or the “wrong” information, that they have had access to, and agreed that it was helpful to have the OT guiding him in his search. Another expressed need was related to the process of selecting appropriate activities—those that fit best with the participant’s desires, interests and abilities. Parents felt anxious (in relation to the safety of the activity) or had preconceptions about the type of activity that was considered “suitable”. To illustrate, with regard to Alex’s desire to learn a new language, his mother reflected how she was unsure what he could choose and assumed the activities would be structured sports groups : “I don’t know anything, but […] I thought it was more, like, sports activities, […], maybe we were confused about what to choose. Leisure? Sports?”. Finally, parents’ need for reassurance was met through the intervention, particularly when parents were concerned or anxious about their child participating in certain activities. For example, one of the biggest obstacles for David’s mother was to let her son take the bus alone in order to achieve his goal of going to the mall. She alluded to the “push” she needed in order to encourage her son to gain autonomy. The OT supported the parents in the process of encouraging their child to become more independent in the community by overcoming some barriers related to their anxiety and reluctance. 

Acknowledging parental needs was inextricably linked to the role of the OT as a guiding figure who assisted in finding activities and facilitating the youth’s independence within the family as well as in the community. This process involved ongoing feedback from the therapist whereby parents felt supported. As David’s mother stated: “You know I was lost, cause there was no one to guide me, no one at the [Health Service Organization] to guide me, you know, so it was really a good thing that she came into our lives.”. The OT was able to find appropriate activities by getting to know the youth and evaluating the feasibility of the activity, for example by going to the facility in which the activity occurred (e.g., the swimming pool). While parental needs were met, a few parents still expressed the desire for ongoing support following the intervention to ensure sustainability of the general process and to maintain participation in community activities as the youth gets older. Some parents expressed the need to have a relationship with the therapist that goes beyond the scope of the study, to be more than just a participant in a research project. As they said: “I wish that we could continue, that we may have a little help to continue” and “I don’t want [the OT] to completely disappear…It’s good to have some support”. Five families however indicated that after the intervention ended, they would continue the efforts made in order to participate in their child’s chosen activities or to find new ones that would fit with their interests. In fact, Sarah’s mother asserted that her daughter will continue engaging in community activities and that Sarah’s participation “is not over”. 

## 4. Discussion

This qualitative study sought to elicit an additional perspective, generated by parents, about the usefulness of the PREP intervention—an approach that has demonstrated effectiveness in improving youth performance in community activities [14,15,17] and was perceived positively by the therapists who delivered it [16]. 

Parents identified a range of benefits associated with the care received, indicating a process of growth extending beyond the accomplishment of the selected activity targeted in the intervention. It involved improvements at the physical, emotional and social levels as well as in their child’s autonomy—all outcomes that were not purposefully targeted by the intervention. While previous research, relying predominantly on cross-sectional studies, also indicated that social participation is positively associated with various youth-related outcomes [6,7,21], this is the first study, to our knowledge, that suggests that increased participation in a meaningful activity can lead to a number of health-related benefits post-intervention. Illustrating the various benefits generated by one single intervention, as is done in this study, can facilitate the development of efficient participation-focused therapies, thereby improving overall health. These findings may be of interest to policy makers and can potentially contribute to improvements in the provision of pediatric rehabilitation services as well as on a broader community level by promoting healthy lifestyle behaviors and ensuring accessibility of activities/services for all children. Further cost-effectiveness studies are however required to test the PREP approach while monitoring the specific benefits or outcomes that emerged in this study. 

The role of the OT was another important finding as it particularly addressed parental needs. Parallels can be drawn between the way parents viewed the role of the OT in this study and the way it was perceived by the very same therapists who delivered the PREP in a recent qualitative study [16]. The role of the OT, from both perspectives, was seen as innovative, where the therapist is a guiding figure and a key facilitator. Both parents and therapists emphasized the advantage of an intervention that is delivered in the participants’ community/environment. These findings further support the call for adopting therapeutic approaches in pediatric rehabilitation that are context-based and involve real-life experiences [22,23], which are currently not well integrated in practice [24]. This is of particular importance as goal-oriented, self-directed therapy approaches that fit with clients’ interests, needs, and capabilities and are implemented in their natural environment, as in this study, are considered recommended practice [25]. 

The fact that the OT identified and selected specific activities together with the youth can be considered a pivotal element of the intervention, and it addressed some parental needs. Interestingly, parents required assistance accessing information and resources about activities and programs as well as in selecting the appropriate type of activity. This suggests that providing information about resources may not be sufficient since parents, together with their child, still need guidance when choosing the just right activity or in setting the most appropriate goal—one that is based on the child’s interests, abilities and existing programs. Indeed, goal setting is an important element of the therapy process and was found to be an effective part of promoting functional outcomes [26] and in increasing parents’ competence and sense of partnership with service providers [27]. 

While overall the intervention had a positive effect on the family, parents’ comments revealed that some needs were not entirely met. Some parents felt they had gained tools and others indicated the need for on-going support. This illustrates the large variety of needs across families, due in part to differences in strengths, values and support systems. As professionals, providing family-centred practice and tailoring our interventions to the unique circumstances and context of each family is key. Considering the range of needs and the complexity of the outcome of participation, the present research emphasizes the importance of offering flexible intervention protocols and customized care to families, in the clinical setting. 

There are some limitations to this study. It conveniently drew on a sample of parents of 12 participants out of 22 families who enrolled in the PREP intervention from only one geographic location. However, this sample size allowed us to reach saturation and was relatively diverse in terms of youth functional issues, family contexts and type of chosen/targeted activities. While certain steps were taken to increase credibility of and confidence in the findings, member checking was not performed due to time constraints, and this may have had an impact on the results. While the majority of the interviewers were independent of the intervention and interviewees remained anonymous, it may be that parents’ comments were framed in a positive light, feeling grateful for the services they received. Finally, this study focused on the parents’ perspective; it is equally important, however, to echo the youth’s voice in further studies in order to obtain a more comprehensive understanding of the experience and the effects of this intervention. 

## 5. Conclusions

Findings on parents’ perspectives provide an additional piece of evidence for the usefulness of the PREP intervention for both youth with disabilities and their families. Such knowledge may assist practitioners in appraising the health-related benefits of participation-based interventions. It can also contribute to the provision of pediatric rehabilitation services by increasing therapeutic options and pathways to positive, health-promoting participation.

## Figures and Tables

**Table 1:** Sample characteristics of the interviewed parents.

Characteristics	*n*
Gender	
*Female (Mother)*	10
*Male (Father)*	2
Community/area of living	
*Suburban area*	6
*Urban center*	4
*Small town*	2
Primary language	
*French*	5
*English*	3
*Arabic*	2
*Spanish*	1
*Bulgarian*	1
Education	
*Graduated university or college*	10
*High school or less*	2
Occupation	
*Full-time working*	4
*Part-time working*	2
*Full-time caregiving*	2
*Recovering from illness*	2
*Studying/looking for a job*	2
Income (per year)	
*Under 10,000$*	1
*20,000–59,999$*	4
*60,000 and 99,999$*	4
*Above 100,000$*	2
Number of children living in the household	
*1*	1
*2*	6
*3*	3
*Not specified/Missing data*	2
